# The New Medical Practice Act

**Published:** 1907-03

**Authors:** 


### THE NEW MEDICAL PRACTICE ACT.

In this issue I publish the famous "One Board Bill," so much discussed and written about, as it finally passed the House today, March 14th. It came out of the Senate some days ago so much amended that its parents scarcely recognized it, but realizing that it was the very best that could be done, the legislative committee and other members of the State Association concluded to let it go at that, and instead of introducing the original bill in the House, they introduced the bill as it came out of the Senate, to avoid fighting it all over again.

The vote in the Senate on the Christian Science exemption amendment was a tie, the Lieutenant Governor (Davidson) having the deciding vote, which killed it. In the House it was killed by 62 to 28.

But accepting the bill as amended in the Senate did not prevent a fight in the House, notwithstanding the pathies all stood in with our men; for the Christian Science and the magnetic healers contested every inch. Amendment after amendment was voted down after hours of wind-jamming, covering the greater part of three days. A strong lobby of physicians and pathies was present three days, but today, on the final fight and passage, the only representative of the State Association present was the editor of the "Red Back," who did all in his power to induce members to pass the Senate bill without the amendment of a single word. Any amendment would have sent the bill back to the Senate and joint conference and certain defeat. I should except Dr. Holman Taylor, the new Assistant State Health Officer, who "did yeoman service."

The bill is defective, vulnerable, and objectionable. It is not what is needed, but it is a big improvement on the Three Board Monstrosity now the law; nor is it what we wanted. But it is the very best possible, and we bow to the wisdom of the majority, who saw safety only in compromise with the other so-called "schools." Luckily, it does not involve recognition of them by the State Association, as it is made the duty of the Governor to mix the board and the Association—in the bill agreed apon—did not ask to be put in affiliation with those who by our Principles of Ethics are "not in honorable standing." The bill now awaits the Governor's signature to become a law. Should he sign it, it goes into immediate effect, repealing the Three Board law. The president of the State Medical Association will have to assemble the House of Delegates at once and submit the required list of names, and the State associations of all the pathies will have to send in their lists, so that the Governor may appoint the board by April 8th, "ninety days after inauguration."

It is thought that the Tuberculosis Sanitarium bill and the Anatomical bill are safe.

